# Involvement of Hormone Receptors, Membrane Receptors and Signaling Pathways in European Gastric Cancers Regarding Subtypes and Epigenetic Alterations: A Pilot Study

**DOI:** 10.3390/biomedicines13081815

**Published:** 2025-07-24

**Authors:** Cynthia Pimpie, Anne Schninzler, Marc Pocard, Véronique Baud, Martine Perrot-Applanat

**Affiliations:** 1 INSERM U1275, Lariboisiere Hospital, Université Paris Cité, 75010 Paris, France; cynthia.crocheray@inserm.fr (C.P.); marc.pocard@inserm.fr (M.P.); 2 Department of Genetics, Pharmacogenomics Unit-Institut Curie, University of Paris Cité, 75005 Paris, France; anne.schninzler@curie.fr; 3 Department of Digestive and Oncology Surgery and INSERM U1275, Pitié Salpetriere Hospital, Université Paris Cité, 75010 Paris, France; 4 NF-κB, Differentiation and Cancer, Faculty of Pharmacy, Université Paris Cité, 75006 Paris, France; veronique.baud@inserm.fr

**Keywords:** gastric cancers (GCs), diffuse gastric subtype, intestinal gastric subtype, hormone receptors, membrane receptors, epigenetics

## Abstract

**Background**: Gastric cancer (GC) is a highly heterogeneous disease and remains one of the major causes of cancer-related mortality worldwide. The vast majority of GC cases are adenocarcinomas including diffuse and intestinal GC that may differ in their incidence between Asian and non-Asian cohorts. The intestinal-subtype GC has declined over the past 50 years. In contrast to the intestinal-subtype adenocarcinoma, the incidence of diffuse-subtype GC, often associated with poor overall survival, has constantly increased in the USA and Europe. The aim of this study was to analyze the expression and clinical significance of steroid hormone receptors, two membrane-bound receptors (*ERRγ* and *GPER*), and several genes involved in epigenetic alterations. The findings may contribute to revealing events driving tumorigenesis and may aid prognosis. **Methods**: Using mRNA from diffuse and intestinal GC tumor samples, the expression level of 11 genes, including those coding for sex hormone receptors (estrogen receptors *ERα* and *ERβ*), progesterone receptor (*PR*) and androgen receptor (*AR*), and the putative relevant *ERRγ* and *GPER* receptor were determined by RT-qPCR. **Results**: In diffuse GC, the expression of *ERα*, *ERβ*, *PR* and *AR* differed from their expression in the intestinal subtype. The expression of *ERα* and *ERβ* was strongly increased in the diffuse subtype compared to the intestinal subtype (×1.90, *p* = 0.001 and ×2.68, *p* = 0.002, respectively). Overexpression of *ERα* and *ERβ* was observed in diffuse GC (15 and 42%, respectively). The expression levels of *PR* and *AR* were strongly decreased in the intestinal subtype as compared to diffuse GC (×0.48, *p =* 0.005 and ×0.25, *p* = 0.003, respectively; 37.5% and 56% underexpression). *ERα*, *ERβ*, *PR* and *AR* showed notable differences for clinicopathological correlation in the diffuse and intestinal GC. A significant decrease of *ERα*, *ERβ*, *PR* and *AR* in intestinal GC correlated with the absence of lymphatic invasion and lower TNM (I-II). In diffuse GC, among the hormone receptors, increases of *ERs* and *PR* mainly correlated with expression of growth factors and receptors (*IGF1*, *FGF7* and *FGFR1*), and with genes involved in epithelial-mesenchymal transition (*VIM* and *ZEB2*) or cell migration (*MMP2*). Our results also report the strong decreased expression of *ERRγ* and *GPER* (two receptors that bind estrogen or xenoestrogens) in diffuse and intestinal subtypes. **Conclusions**: Our study identified new target genes, namely hormone receptors and membrane receptors (*ERRγ* and *GPER*), whose expression is associated with an aggressive phenotype of diffuse GC, and revealed the importance of epigenetic factors (*EZH2*, *HOTAIR*, *H19* and *DNMT1*) in gastric cancers.

## 1. Introduction

Gastric cancer (GC) is the fifth leading cause of cancer-related death [1,2,3], and is highly heterogeneous between different subtypes according to the classification proposed by the World Health Organization [4]. The vast majority (about 95%) of gastric tumors are adenocarcinomas, which can be further histologically classified into intestinal, diffuse and mixed types according to the Lauren classification [5]. Intestinal GC is well differentiated and related to the *Helicobacter pylori* infection clustered subtype with a decreased incidence in Eastern Asia [6]. On the contrary, the incidence of diffuse GC is increasing worldwide, especially in Western countries (Europe and the USA) (e.g., 0.1 to 1.4/year for 100,000 inhabitants between 1973 and 2000 in the USA) [7,8]. Most patients with diffuse GC are diagnosed at an advanced stage, with lymphovascular invasion, peritoneal carcinomatosis and poor prognosis. These patients are generally refractory to conventional therapeutic approaches [9,10]. Recent efforts investigate the cellular and molecular mechanisms leading to the development and progression of diffuse GC. Our group has previously identified several genes whose expression is associated with the aggressive phenotype of diffuse GC [11,12,13].

Sex hormone receptors such as estrogen receptors (*ERα* and *ERβ*), progesterone receptor (PR) and androgen receptor (AR) are members of the hormone receptor family and have been associated with several human cancers, including breast, ovarian, prostate, colon, pancreas and hepatocellular carcinoma [14,15]. A few studies, mainly from Asian groups, have reported the expression levels of hormone receptors (ERs, PR and AR) in gastric cancers [15,16,17,18]. However, the distribution of these receptors in different GC subtypes and any correlations of their expression levels with clinical parameters are currently unknown. The repertoire of estrogen receptors has been expanded and now includes *ERRγ* (estrogen receptor-related receptor) and *GPER* (G protein-coupled estrogen receptor). These receptors are membrane-bound receptors that have been poorly investigated in gastric cancers. *ERRγ* and *GPER* have been considered to play a role as modulators of estrogen signaling, especially in breast cancers [19,20,21,22,23]. However, their distribution in GC subtypes and their functions are still currently unknown.

In this study, with mRNA from gastric tumors and normal gastric tissues, we used real-time RT-qPCR assays to assess the expression of mRNA of four sex hormone receptors and two membrane receptors in gastric tumors and normal gastric tissues. We compared the expression of each hormone receptor with clinicopathological parameters of each subpopulation of GC. We also analyzed the expression of genes involved in epigenetic factors, such as EZH2 (Enhancer of zest homolog 2), involved in histone modifications, and HOTAIR (a long non-coding RNA), two genes that have been associated with tumorigenesis in several cancers. We finally examined the correlation of expression between hormone receptors, membrane receptors and genes involved in epigenetic mechanisms.

## 2. Materials and Methods

### 2.1. Patients and Tissue Samples

Our cohort of gastric cancers (including diffuse and intestinal subtypes) was previously described [11,12,13]. A total of 29 patients underwent partial gastrectomy for histopathologically confirmed gastric adenocarcinoma primary tissue in the Lariboisiere Hospital (Paris, France) from 2005–2014. All patients provided written informed consent prior to their inclusion in the study [11,12,13]. The population was divided into 2 groups according to the histological status of GC: intestinal subtype (n = 16) or diffuse subtype (n = 13) according to the Lauren classification. The malignancy of infiltrating carcinomas was scored according to TNM staging system (Stage I to IV). The median age of patients with diffuse GC was significantly lower [57 (27–71) years] as compared with patients with the intestinal subtype [75 (59–82) years] (*p* = 0.0004). Patients with diffuse GC are younger and exhibit more aggressive characteristics (more lymphatic invasion, 85% *p* = 0.0014, and massive stromal fibrosis) than patients with the intestinal subtype.

### 2.2. Total RNA Preparation and RT-qPCR

The conditions for total RNA extraction, complementary cDNA synthesis and qPCR conditions were as described elsewhere [24,25] using an ABI Prism 7900 Sequence Detection System (Applied Biosystems, Thermo Fisher Scientific, Inc., Waltham, MA, USA). Primers for hormone receptors and other genes were selected using Oligo 6.0 (National Biosciences, Plymouth, MN) [12,25]. Each sample was normalized on the basis of 3 endogenous RNA control genes involved in various cellular metabolic pathways, namely *TBP,* which encodes the TATA-box binding protein; *RPL0*, which encodes human acidic ribosomal phosphoprotein P0; and *PPIA*, which encodes peptidylprolyl isomerase 2 (also known as cyclophilin), as previously described [11,25]. Results, expressed as N-fold difference in target gene expression relative to the *TBP* gene (and termed “Ntarget”), were determined as the Ntarget = 2^ΔCtsample^, where the ΔCt value of the sample was determined by subtracting the average Ct value of the specific target gene from the average Ct value of the *TBP* gene. The Ntarget values of the samples were subsequently normalized so that the median of the Ntarget values for normal gastric tissues (n = 11) was 1. The target gene expression was normalized to its transcription level of housekeeping genes *TBP* and peptidylprolyl isomerase 2 (*PPIA*). Preliminary analysis of gene expression did not indicate changes in median basal levels in normal samples in the same patients (with either diffuse or intestinal GC). For each gene, normalized RNA values of 3 (or more) were considered to represent gene overexpression in tumor samples, and values of 0.33 (or less) represented gene underexpression.

### 2.3. Statistical Analysis

As mRNA expression levels did not fit a Gaussian distribution, the relative expression of genes was characterized by the median and the range rather than by their mean values and coefficient of variation [11,12,25]. For each gene, differences of expression between tumors and normal tissues (fold change) were analyzed using the Mann–Whitney U test as previously described [11,25]. Differences in the number of samples that overexpressed (>3-fold) or underexpressed (<3-fold) were analyzed using the Chi^2^ test [12]. The relationships between expressions of genes in GC were determined using the non-parametric Spearman’s rank correlation test. The relationships between expression levels and clinical parameters were analyzed using the non-parametric Kruskal–Wallis (or Mann–Whitney) and Chi-square tests, as indicated in each Table. Statistical analyses were performed using Prism 5.03 (GraphPad, San Diego, CA, USA). Differences were considered significant at confidence levels greater than 95% (*p* < 0.05).

## 3. Results

### 3.1. Expression Profiles of Sex Hormone Receptors ERα, ERβ, PR and AR in Gastric Cancers

**Figure 1:**
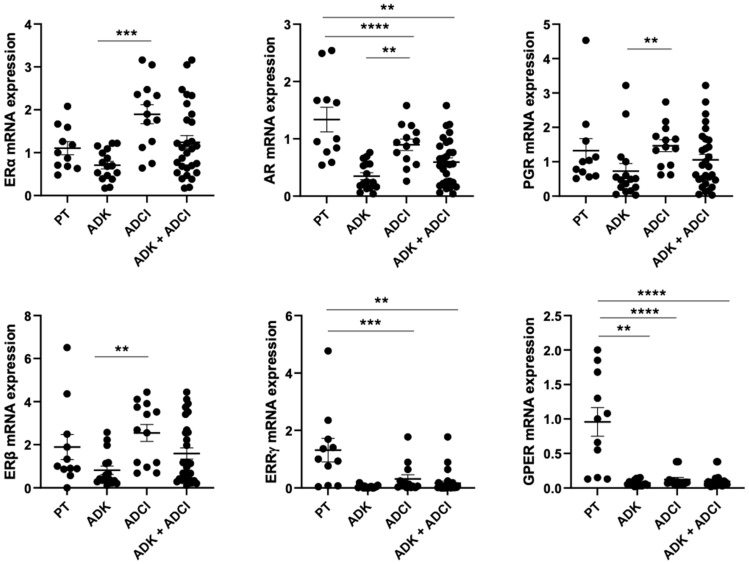
mRNA expression levels of steroid receptors and membrane receptors (*ERRγ* and *GPER*) in non-tumoral tissue (PT), diffuse and intestinal GC. ** *p* value < 0.01; *** *p* value < 0.001; **** *p* value < 0.0001.

Real-time qPCR was used to analyze the expression of *ERα*, *ERβ*, *PR* and *AR* in non-tumoral and tumoral GC subtypes. As compared with normal gastric tissues (PT), the expression of *ERα*, *ERβ* and *PR* was unchanged in all gastric tumors (Table 1). In diffuse-subtype GC (Table 1 and Figure 1) the expression of *ERα* was significantly increased (X1.9, *p* = 0.01). The expression of *ERβ* was also increased (X2.68) with 46% overexpression compared to normal mucosa (Table 1 and Supplementary Table S1A). The expression levels of *PR* and *AR* were not significantly changed (Table 1). In intestinal-subtype GC (Table 1 and Figure 1), the expression of *ERα* (×0.7, *p =* 0.04), *ERβ* (×0.4, *p* = 0.06), *PR* (×0.48, *p* = 0.013) and *AR* (×0.25, *p* <0.0001) were significantly decreased as compared to normal mucosa, along with underexpression of *ERα* (12%), *ERβ* (31%), *PR* (37%) and *AR* (56%) (Supplementary Table S1B). Altogether, comparison of steroid receptors in subtypes of GC revealed significantly higher expression levels of *ERα* (*p* = 0.0005), *ERβ* (*p* = 0.002), *AR* (*p* = 0.003) and *PR* (*p* = 0.005) in diffuse GC as compared to intestinal-subtype GC (Figure 1).

### 3.2. Relationship Between Hormone Receptor Expression and Clinical Parameters in Gastric Cancers Including Diffuse and Intestinal GCs

Clinicopathological characteristics included sex, age, tumor grade, vascular and lymphatic invasion and TNM classification. In all tumors, the higher expressions of *ERα*, *ERβ*, *PR* and *AR* were observed in younger patients (<60 years), and in patients with lymphatic invasion and higher TNM (III–IV). In contrast to diffuse subtype (Table 2A), low expression of *ERα*, *PR* and *AR* in intestinal GC was significant in patients without lymphatic invasion and lower TNM (I–II) (Table 2B).

### 3.3. Correlations Between the Expression of Sex Hormone Nuclear Receptors and Signaling Pathways in GCs

To go one step further, we then compared the expression of sex hormone receptors with signaling pathways including proliferation, EMT and migration as previously described [11]. In diffuse GC (Table 3), the expression of *ERα* significantly correlated with *PR* (*p* = 0.001) and *AR* (*p* = 0.02), but not *ERβ* (*p* = 0.07). The increased expression of *ERα* and *PR* correlated with several genes encoding growth factors and their receptors including *IGF1* (*p* < 0.02), *FGF7* and *FGFR1* (*p* < 0.001), genes involved in EMT such as *VIM* (*p* = 0.001), *ZEB2* (*p* < 0.001), *Slug* (*p* = 0.007), *CXCL12* (*p* < 0.001) and in migration such as *MMP2* (*p* < 0.01) (Table 3). Increased expression of *ERβ* significantly correlated with growth factors such as *FGF7* and *FGFR1* (*p* = 0.02). *AR* expression also correlated with *FGFR1* (*p* = 0.02) and *CXCL12* (*p* = 0.005) (Table 3).

In intestinal GC (Table 4), significantly decreased expression of *ERα*, *PR* and *AR* compared to non-tumoral tissue showed correlations with growth factors (*IGF1* (*p* < 0.0001), *IGFR1* (*p* = 0.015), *FGF7* (*p* = 0.0001) and *FGFR1* (*p* = 0.002 for *ERα*, *p* < 0.0001 for *PR* and *AR*), genes involved in EMT including *VIM* (*p* = 0.02) for *ERα*, *p* < 0.002 for *PR, p* = 0.0002 for *AR*), *ZEB2* (*p* = 0.0001 for *ERα* and *AR*, *p* = 0.004 for *PR*) (Table 4). Moreover, the decrease of *ERα* (*p* = 0.003), *PR* (*p* = 0.0001) and *AR* (*p* < 0.0001) was inversely correlated with *MKI67* and *p53* expression (Table 4).

### 3.4. Expression of Two Membrane Receptors, ERRγ and GPER, in Gastric Cancers

We further analyzed the expression of two members of membrane steroid hormone receptors, *ERRγ* (estrogen-related receptor) and *GPER/GPR30* (G protein-coupled estrogen receptor) that are closely related to the steroid hormone receptor family, revealing clinically relevant associations. *ERRγ* is an orphan receptor closely related to the estrogen receptor family. These receptors are known to mediate rapid, non-genomic effects of estradiol in hormone-related cancers and in many cell types. In all GCs, as compared to normal gastric tissues, the mRNA expression levels of *ERRγ* and *GPER* were significantly decreased (×0.04, *p* = 0.001 and ×0.07, *p* = 0.0001, respectively) (Table 1). No difference in the expression of *ERRγ* or *GPER* was observed between the diffuse and intestinal subtypes (Table 1 and Figure 1). Significant underexpression of *ERRγ* and *GPER* was also observed in diffuse or intestinal subtypes, as compared to normal tissue (*p* = 0.0002) (Supplementary Table S1A,B). The expression of *ERRγ* is lower in male patients with diffuse GC (*p* = 0.001, Table 2A). *GPER* expression was also decreased in GC subtypes, along with 85% and 100% underexpression in diffuse or intestinal GC (Table 1 and Figure 1). However, low *GPER* expression significantly correlates with the absence of lymphatic invasion (*p* < 0.02) and TNMI-II (*p* < 0.03) in patients with intestinal GC (Table 2B).

We also compared the expression of *ERRγ* and *GPER* with genes coding for hormone receptors and genes involved in signaling pathways including proliferation, EMT and migration in GC subtypes (Table 5). In intestinal GC (Table 5B), but not in diffuse GC (Table 5A), low expression of *GPER* was significantly correlated with the expression of steroid receptors, growth factors, genes involved in EMT (*ZEB2*, *CXCL12*), and was inversely correlated with *MKI67* and *p53* (Table 5). The low expression of *ERRγ* in intestinal GC was inversely correlated with *p16* and *AhR* (Table 5B).

### 3.5. Expression of Epigenetic Marks/Factors in GCs

Several studies have revealed the importance of epigenetic gene regulation as central to tumorigenesis (tumor progression and metastasis) in several cancers including gastric cancers [26,27,28]. We further analyzed in gastric cancer subtypes the expression of genes involved in epigenetic controls, such as *EZH2* (Enhancer of zest homolog 2 involved in histone modifications), non-coding RNA (including long non-coding RNAs such as *HOTAIR*, *H1*9) and *DNMT1* (involved in DNA methylation). As measured by qPCR, the expression of *EZH2* was significantly increased in all tumors (×3, *p* < 0.0001), intestinal and diffuse GCs (Table 1 and Figure 1), along with significant overexpression (75% and 23%, respectively, *p* = 0.016) in GC subtypes (Supplementary Table S1A,B). *EZH2* expression depends on clinical parameters such as age, tumor and lymphatic invasion, TNM and metastasis in all tumors, with differences within subtypes (Table 6). Notably, higher *EZH2* expression was observed in the oldest patients with diffuse GC, and in the absence of lymphatic invasion and in TNMI-II in intestinal GC (Table 6A,B). The significant increase of *EZH2* expression observed in intestinal GC was inversely correlated with the expression of *ERα*, *PR* and *AR* (*p* = 0.04), *GPER* (*p* = 0.001) and *P53* (*p* = 0.004) (Table 4 and Table 5).

The expression of the lncRNA *HOTAIR* was significantly increased in gastric tumors (×19.2, *p* = 0.0002) (Table 1), both in diffuse (×8.4, *p* = 0.02) and intestinal (×20.8, *p* < 0.001) GC subtypes. High *HOTAIR* expression was significantly observed in early stages (T1-T2, *p* = 0.01), and in later stages (TNM III-IV, *p* = 0.03) in diffuse GC (Table 6). Furthermore, the expression of *DNMT1* was significantly increased in all tumors, especially in intestinal GC (Table 6B). An increase of *H19* (another lncRNA) was observed in all tumors, with overexpression (>3) in diffuse and intestinal GC (46% and 56%, respectively) (Table 1 and Supplementary Table S1A,B). The expression of *BRCA1*, a gene involved in DNA repair, was significantly increased in all tumors (×2.4, *p* < 0.0001), both in diffuse and intestinal GC (Table 1), along with 56% overexpression (>3) in the intestinal subtype.

## 4. Discussion

We and others previously reported that diffuse GC is an aggressive and infiltrating carcinoma with a substantially increasing incidence in Europe and the USA [7,8]. Diffuse GC is usually diagnosed at an advanced stage (lymphatic invasion and peritoneal carcinomatosis) [11,12,13], contributing to its poor prognosis and major obstacles to therapy [29,30,31]. Although hormone receptors have been reported in gastric cancers in Eastern studies, their expression and role in GC subtypes remain unexplored in Western patients. In the present study we report for the first time the elevated expression levels of steroid hormone receptors such as *ERα* and association with proliferation, epithelial mesenchymal transition and migration in diffuse GC. We also report for the first time the significantly decreased expression of membrane receptors (*ERRγ* and *GPER*) in GC subtypes, suggesting their role as tumor suppressor genes. Furthermore, we describe expression level differences for genes involved in epigenetic alterations such as histone modification (EZH2), long non-coding RNA (lncRNA, such as HOTAIR and H19) and DNA methylation in GCs. Thus, the analysis of tumor biomarkers implicated in initiation and progression in GCs may represent a new approach for further therapeutic management of patients with diffuse GC.

Expression of hormone receptors. Interactions of sex hormones with their receptors are frequently agonistic in the carcinogenic process. Several studies have reported the expression and role of hormone receptors in several cancers, such as breast, ovary, endometrium, testis, pancreas and liver [14,15,17,32,33]. However, little is known about the involvement of sex hormone receptors in GCs, such as whether they are specific for a subpopulation of GC, or if they are relevant for prognosis [16,17,18,34,35,36]. Using our well-described cohort [11,12,13], we explored the expression of sex hormone receptors in diffuse and intestinal GCs, as well as the mechanisms that underlie their expression and signaling pathways (association with development and progression). We show for the first time a higher expression of *ERα*, *ERβ*, *PR* and *AR* in diffuse GC, as compared to intestinal GC, and the significant correlation in diffuse GC of *ERα* and *PR* with growth factor genes (*IGF1*, *FGF7* and *FGFR1*), genes involved in the epithelial-mesenchymal transition (EMT, among which *VIM*, *ZEB2* and *SLUG*) and in cell migration (*MMP2*). The IGF system promotes cancer proliferation and induces the EMT phenotype, which contributes to the migration, invasiveness and metastasis of diffuse GC [11]. We also highlight the increased expression of *ERβ* and the identification of the *ERβ*-regulated gene network through *FGF7* and *FGFR1* in diffuse GC. Overexpression of *FGF* and *FGFR1* has been reported in multiple cancers associated with lymphovascular invasion, distant metastasis and poor survival [37,38]. Altogether, our results suggest that the increased levels of hormone receptors in diffuse GC are associated with invasion, metastasis and an unfavorable prognosis. In contrast, the significant low expression of *ERα*, *PR* and *AR* in intestinal GC inversely correlated with *p53*, a recognized tumor suppressor that prompts cell cycle arrest and apoptosis.

Expression of membrane receptors. In the present study, we also investigated the expression and clinical relevance of *ERRγ* and *GPER*, two membrane hormone receptors that play important roles in hormonally responsive cancers [23,39]. Our results indicate a significant decrease in both *ERRγ* and *GPER* expression in diffuse and intestinal GCs as compared to the level in non-tumoral gastric mucosa. In diffuse GC, the decrease of *ERRγ* correlated with sex and increased expression of *MMP2*. *ERRγ* is a key regulator of cellular metabolism (ion homeostasis) in the highly oxidative gastric mucosa [39,40], promotes mesenchymal-to-epithelial transition and inhibits the growth of tumor xenografts [21]. More recently, *ERRγ* has been described as a tumor suppressor that inhibits Wnt signaling in GC and is a predictor of poor clinical outcome [41].

We also observed a decrease of *GPER* expression in gastric cancer, in both diffuse and intestinal subtypes (*p* < 0.0002 and *p* < 0.0001, respectively) as compared to the level of expression in normal gastric mucosa. *GPER* underexpression (100%) in intestinal GC was significantly associated with the absence of lymphatic invasion and TNMI-II. Downregulation of *GPER* has been previously shown in various types of cancers including breast, ovarian, lung and colon cancers [42,43], and is also associated with an unfavorable factor for overall survival [44,45,46]. *GPER* has emerged as a tumor suppressor in cancer [47]. Altogether, our results suggest that *ERRγ* and *GPER* could play a role as tumor suppressors in diffuse and intestinal GCs.

While *ERRγ* has long been considered as an orphan nuclear receptor closely related to the estrogen receptor (ERR) family, it is now considered that estradiol and xenoestrogens (endocrine disrupting chemicals including bisphenol A, BPA) activate *ERRγ* and *GPER* with a strong affinity. *ERRγ* and *GPER* have been considered to be involved in the rapid BPA-dependent activation of intracellular signaling [20,48,49,50,51,52,53,54]. Several studies have also indicated that BPA and environmental estrogens can stimulate epithelial and stromal transcriptome, promoting cancer progression [51,53,55]. Whether the different expressions of hormone receptors that we observed in diffuse and intestinal GC are due to long exposure to a specific or multiple environmental substances remains to be established in diffuse GC.

Expression and roles of epigenetic factors in gastric cancer subtypes. Epigenetic alterations are driver events in tumorigenesis, regulating tumor progression, metastasis and resistance to therapy through EMT [56]. *EZH2* and *HOTAIR* (an oncogenic long non-coding RNA) were found to be expressed at significantly high levels in intestinal and diffuse GCs compared to normal tissues. EZH2 is a histone methyltransferase responsible for the trimethylation of histone H3 (H3K27), closely associated with gene silencing in development and a molecular marker for a precancerous state [57,58]. Overexpression of *EZH2* is related to a poor prognosis in digestive cancers [59]. A significant inverse correlation between expression of *GPER* and *EZH2* was observed in intestinal GC (*p* = 0.001), suggesting that *GPER* expression could be downregulated by epigenetic regulation.

In our study, the expression of *HOTAIR* was significantly increased in diffuse GC (*p* = 0.02), where it is associated with early tumor invasion, higher TNM and metastasis and in intestinal GC (*p* < 0.0001). HOTAIR contributes to GC development and induces the EMT transition in GC cells [60,61]. A high expression of *HOTAIR* has been associated with advanced pathological stage, tumor progression and increased metastasis, and is an indicator of a poor prognosis and resistance to chemotherapy [62,63,64,65,66,67,68]. HOTAIR regulates the proliferation, colony formation, migration and self-renewal capacity of cancer stem-like cells [66]. Several studies have shown that HOTAIR reprograms chromatin to promote cancer metastasis and transcriptional silencing of metastasis suppressor genes [69,70]. HOTAIR interacts with EZH2 and is upregulated in a variety of cancers (breast, lung, colon and gastric) [71]. Moreover, the expression of EZH2 and HOTAIR have been found to be regulated by estradiol or environmental endocrine disrupting chemicals in several cancers and in vitro [53,72,73,74,75,76,77].

We also show overexpression (>3) of H19 (a lncRNA) in diffuse and intestinal GCs (Table 1). Abundant expression of H19 is found in human cancers, including breast, ovary, colon and hepatocellular cancers and GC. H19 acts as an oncogene, regulates gene expression and plays a significant role in E2-induced proliferation in breast cancer cells [78]. H19 is implicated in cancer progression (proliferation, migration, invasion and metastasis) [79], as well as endocrine therapy resistance [78], contributing to poor prognosis [80]. DNMT1 is responsible for the maintenance of methylation patterns throughout DNA replication.

## 5. Conclusions

This pilot study explores two forms of GCs, diffuse and intestinal, which lead to metastasis in the peritoneal cavity. The increase of sex hormone receptors, their expression associated with growth, the epithelial-mesenchymal transition and migration, and aggressiveness may potentially serve as biomarkers for exposure to endogenous or exogenous endocrine disruptors in diffuse GC. The decrease of membrane receptors *ERRγ* and *GPER* is associated with growth, epithelial-mesenchymal transition and migration, and aggressiveness in leading to metastasis in the peritoneal cavity. Moreover, the increased expression of *EZH2*, *HOTAIR* and *H19* in gastric subtypes may result in tumorigenesis and metastasis and predict a poor prognosis. Taken together, applications of our findings could involve hormone antagonists and molecules involved in small extracellular vesicles. We acknowledge that our study has some limitations. Because of the small number of tumor samples, the results need to be confirmed using a larger cohort of patients with gastric cancers. Subsequent in vitro studies will also provide a better understanding of the complexity of epigenetic mechanisms. Nonetheless, our pilot study presents evidence that tumor biomarkers such as steroid receptors, membrane receptors and epigenetic factors represent a new approach to discriminate between the diffuse and intestinal GC subtypes, to act as indicators of a poor prognosis and to guide therapy in diffuse GC.

## Figures and Tables

**Table 1:** Statistical analysis of mRNA expression levels of steroid receptors, membrane receptors and genes involved in epigenetics in gastric cancers (all GC, diffuse (ADCI) and intestinal (ADK) GC.

Genes	PT (n = 11)	Tumoral (n = 29)	*p*-Value ^a^	ADCI (n = 13)	*p*-Value ^a^	ADK (n = 16)	*p*-Value ^a^	ADCI vs. ADK *p*-Value ^b^
***A,* Receptors**								
***ERα***	1 (0.48–2.08)	1.12 (0.17–3.16)	>0.99 (NS)	1.90 (0.64–3.16)	**0.01**	0.69 (0.17–1.22)	**0.04**	**0.0005**
***ERβ***	1 (0–6.51)	1.13 (0.12–4.44)	>0.99 (NS)	2.68 (0.68–4.44)	0.15 (NS)	0.40 (0.12–2.57)	0.06 (NS)	**0.002**
***AR***	1 (0.54–2.54)	0.56 (0.04–1.58)	**<0.01**	0.94 (0.26–1.58)	0.17 (NS)	0.25 (0.04–0.76)	**<0.0001**	**0.003**
***PR***	1 (0.51–4.53)	0.86 (0.03–3.22)	>0.99 (NS)	1.43 (0.62–2.74)	0.20 (NS)	0.48 (0.03–3.22)	**0.013**	**0.005**
***ERRγ***	1 (0.04–4.77)	0.04 (0.00–1.77)	**0.001**	0.07 (0.01–1.77)	**0.011**	0.02 (0.00–0.18)	**0.0002**	0.25 NS
***GPER***	1 (0.13–2)	0.07 (0.02–0.38)	**<0.0001**	0.08 (0.04–0.38)	**<0.0001**	0.06 (0.02–0.15)	**<0.0001**	0.99 (NS)
***B,* Epigenetic**								
***EZH2***	1 (0.21–2.19)	3.02 (1.11–9.45)	**<0.0001**	2.51 (1.19–3.81)	**0.03**	4.34 (1.11–9.45)	**<0.0001**	0.31 NS
***HoTAIR***	0 (0–3.1)	19.2 (0–67.8)	**0.0002**	8.40 (0–52.6)	**0.02**	20.8 (0.25–67.8)	**<0.0001**	0.99 (NS)
***H19***	1 (0.38–12.3)	3.54 (0.4–42.3)	0.12	2.5 (1.1–42.3)	0.48 (NS)	6.94 (0.4–27.8)	0.12	0.99 (NS)
***DnmT1***	1 (0.77–1.47)	1.6 (0.86–2.5)	**0.0007**	1.5 (1.1–1.75)	0.10 (NS)	2.05 (0.9–2.5)	**0.0001**	0.38 NS
***MALAT***	1 (0.56–2.02)	0.65 (0.24–1.92)	0.11	0.61 (0.26–1.22)	0.09 (NS)	0.69 (0.24–1.92)	0.39	0.99 (NS)
***C,* DNA repair**								
***BRCA1***	1 (0.3–1.70)	2.38 (1.38–6.86)	**<0.0001**	2 (1.47–3.33)	**0.025**	3.12 (1.38–6.86)	**<0.0001**	0.12 (NS)

Median (range) of gene mRNA expression levels in all tumors, ADCI or ADK vs. peri-tumoral tissue. *p* value (**^a^** Mann–Whitney test). Significant *p* value in bold. NS = not statistically different. Comparative level of expression of genes in ADCI vs. ADK (**^b^** Kruskal–Wallis test). These receptors were expressed in normal mucosa with different basal levels (arbitrary median values vs. CT35 were 114 (AR), 48 (*ERα*), 13 (*ERβ*) and 10 (*PR*), respectively.

**Table 2:** Relationship between expression of hormone receptors or membrane receptors and clinical parameters in (**A**) diffuse gastric cancers and (**B**) intestinal gastric cancers.

**(A)**	***ERα***	***ERβ***	***PR***	***AR***	***ERRγ***	***GPER***
**Gender,** **Male n = 6)** **Female (n = 7)**	*p* = 0.23 2.34 (0.6–3.2) 1.71 (0.7–3)	*p* = 0.36 3 (1.2–4.1) 1.2 (0.7–4.4)	*p* = 0.31 1.64 (0.6–2.7) 1.33 (0.6–2)	*p* = 0.73 0.85 (0.5–1.2) 0.96 (0.3–1.6)	***p* = 0.001** 0.02 (0.01–0.06) 0.24 (0.07–1.8)	*p* = 0.11 0.07 (0.04–0.1) 0.09 (0.04–0.4)
**Age** **<60 years (n = 8)** **>60 years (n = 5)**	*p* = 0.62 2.23 (0.6–3.2) 1.76 (0.75–2.5)	*p* = 0.13 3.6 (0.7–4.4). 1.17 (0.7–3.4)	*p* = 0.59 1.5 (0.6–1.97) 1.43 (0.9–2.7)	*p* = 0.52 0.98 (0.3–1.6) 0.76 (0.5–1.1)	*p* = 0.94 0.09 (0.01–0.9) 0.07 (0.01–1.8)	*p* = 0.59 0.08 (0.04–0.4) 0.08 (0.04–0.4)
**Tumor invasion** **T1-T2 (n = 2)** **T3-T4 (n = 11)**	*p* = 0.53 1.73 (1.7–1.8) 2.14 (0.6–3.2)	*p* = 0.18 1.1 (1–1.2) 3.4 (0.7–4.4)	*p* = 0.63 1.16 (0.9–1.4) 1.63 (0.6–2.7)	*p* = 0.51 1.03 (1–1.1) 0.87 (0.3–2.7)	*p* = 0.15 0.15 (0.07–0.2) 0.06 (0.01–1.8)	*p* > 0.999 0.08 0.08 (0.04–0.4)
**Vascular invasion** **negative (n = 3)** **positive (n = 10)**	*p* = 0.57 2.33 (1.1–3.2) 1.83 (0.6–3.2)	*p* = 0.37 3.52 (2.5–4.1) 1.93 (0.7–4.4)	*p* = 0.49 1.38 (0.6–1.7) 1.53 (0.6–2.7)	*p* = 0.94 1.02 (0.3–1.2) 0.90 (0.5–1.2)	*p* = 0.37 0.04 (0.01–0.1) 0.12 (0.01–0.8)	*p* = 0.45 0.07 (0.04–0.1) 0.08 (0.04–0.4)
**Lymphatic invasion** **negative (n = 1)** **positive (n = 12)**	*ND* 2.33 1.83 (0.6–3.2)	*ND* 4.11 3.6 (0.7–4.4)	*ND* 1.38 1.53 (0.62–2.7)	*ND* 2.02 0.90 (0.3–1.6)	*ND* 0.01 0.1 (0.01–1.8)	*ND* 0.07 0.08 (0.04–0.4)
**Peritoneal metastasis** **negative (n = 9)** **positive (n = 4)**	*p* = 0.15 2.14 (0.6–3.2) 1.19 (0.7–2.3)	*p* = 0.55 2.68 (1–4.4) 2.1 (0.7–4.1)	*p* = 0.05 1.66 (0.6–2.7) 1.09 (0.6–1.4)	*p* = 0.60 0.94 (0.5–1.6) 0.78 (0.3–1)	*p* = 0.33 0.06 (0.01–0.6) 0.51 (0.01–1.8)	*p* = 0.68 0.08 (0.04–0.12) 0.22 (0.04–0.4)
**TNM** **I-II (n = 5)** **III-IV (n = 8)**	*p* = 0.22 2.36 (0.6–3.2) 1.73 (0.7–2.5)	*p* = 0.17 3.74 (1.2–4.4) 1.92 (0.7–4.1)	*p* = 0.53 1.66 (0.6–1.97) 1.35 (0.6–2.7)	*p* = 0.52 0.94 (0.5–1.6) 0.86 (0.3–1.2)	*p* = 0.83 0.06 (0.01–0.6) 0.1 (0.01–1.8)	*p* = 0.37 0.09 (0.07–0.12) 0.07 (0.04–0.4)
**(B)**	***ERα***	***ERβ***	***PR***	***AR***	***ERRγ***	***GPER***
**Gender,** **Male (n = 7)** **Female (n = 9)**	*p* = 0.78 0.77 (0.2–1.2) 0.68 (0.4–1.2)	*p* = 0.92 0.38 (0.1–2.2) 0.42 (0.2–2.6)	*p* = 0.78 0.49 (0.1–1.1) 0.47 (0.03–3.2)	*p* = 0.42 0.25 (0.04–0.7) 0.15 (0.1–0.8)	*p* > 0.999 0.04 (0–0.06) 0.15 (0.1–0.8)	*p* = 0.98 0.07 (0.03–0.1) 0.06 (0.02–0.1)
**Age** **<60 years (n = 1)** **>60 years (n = 15)**	ND 1.16 0.7 (0.2–1.2)	ND 2.22 0.4 (0.1–2.6)	ND 0.7 0.2 (0.04–0.8)	ND 1.14 0.5 (0.03–3.2)	ND 0.04 0.02 (0–0.2)	ND 0.14 0.06 (0–0.2)
**Tumor invasion, T** **T1-T2 (n = 4)** **T3-T4 (n = 12)**	*p* = 0.22 0.33 (0.2–1.2) 0.73 (0.4–1.2)	*p* = 0.26 0.25 (0.1–2.6) 0.51 (0.2–2.2)	*p* = 0.21 0.26 (0.1–0.5) 0.53 (0.03–3.2)	*p* = 0.07 0.14 (0.04–0.2) 0.46 (0.1–0.8)	*p* = 0.31 0.05 (0–0.2) 0.02 (0–0.1)	*p* = 0.34 0.04 (0.03–0.1) 0.06 (0.02–0.1)
**Vascular invasion,** **Negative (n = 6)** **Positive (n = 10)**	*p* = 0.19 0.88 (0.5–1.2) 0.61 (0.2–1.2)	*p* = 0.09 0.9 (0.2–2.6) 0.36 (0.1–2.2)	*p* = 0.58 0.48 (0.1–3.2) 0.43 (0.03–1.1)	*p* = 0.54 0.23 (0.1–0.8) 0.31 (0.04–0.7)	*p* = 0.44 0.04 (0–0.02) 0.02 (0–0.07)	*p* = 0.13 0.09 (0–0.2) 0.05 (0–0.01)
**Lymphatic invasion,** **Negative (n = 10)** **Positive (n = 5)**	*p* = 0.009 * 0.50 (0.2–1.2) 1.09 (0.9–1.2	*p* = 0.37 0.37 (0.1–2.6) 0.6 (0.3–2.2)	*p* = 0.003 * 0.26 (0.03–0.6) 1.14 (0.5–3.2)	*p* = 0.03 * 0.17 (0.04–0.6) 0.66 (0.4–0.8)	*p* = 0.74 0.02 (0–0.2) 0.04 (0–0.09)	*p* = 0.017 * 0.05 (0.02–0.1) 0.14 (0.05–0.15)
**Peritoneal metastasis** **negative (n = 15)** **positive (n = 1)**	*ND* 0.68 (0.2–1.2) 1.16	*ND* 0.39 (0.1–2.6) 2.22	*ND* 0.47 (0.03–3.2) 1.14	*ND* 0.24 (0.04–0.8) 0.66	*ND* 0.02 (0–0.2) 0.04	*ND* 0.06 (0–0.1) 0.14
**TNM** **I-II (n = 11)** **III-IV (n = 5)**	*p* = 0.006 * 0.53 (0.2–1.2) 1.09 (0.9–1.2)	*p* = 0.51 0.39 (0.1–2.6) 0.6 (0.3–2.2)	*p* = 0.002 * 0.26 (0.03–0.6) 1.14 (0.5–3.2)	*p* = 0.002 * 0.18 (0.04–0.6) 0.66 (0.4–0.8)	*p* = 0.80 0.02 (0–0.2) 0.04 (0–0.09)	***p* = 0.024** 0.05 (0.02–0.12) 0.14 (0.05–0.15)

Median (range) of gene mRNA expression levels; *p* value (Mann–Whitney). Significant *p* value * in bold. ND = not determined.

**Table 3:** Correlations between the expression of hormone receptors and genes involved in signaling pathways in diffuse GC.

Genes	*ERα*		*ERβ*		*AR*		*PR*	
	r	*p*-Value ^a^	r	*p*-Value ^a^	r	*p*-Value ^a^	r	*p*-Value ^a^
**Hormone receptors**								
***ERα***	**1**	**<0.0001**	0.516	0.07	**0.615**	**0.024**	**0.826**	**0.001**
***ERβ***	0.516	0.07	**1**	**<0.0001**	−0.038	0.90	0.414	0.16
***AR***	**0.615**	**0.024**	−0.038	0.90	**1**	**<0.0001**	0.373	0.21
***PR***	**0.826**	**0.001**	0.414	0.16	0.373	0.21	**1**	**<0.0001**
***ERRγ***	−0.434	0.14	−0.499	0.08	0.099	0.75	−0.370	0.21
***GPER***	−0.011	0.97	−0.311	0.30	0.480	0.09	−0.007	0.98
***AhR***	−0.072	0.81	−0.487	0.09	0.198	0.52	0.134	0.66
**Growth factors**								
***IGF1***	**0.654**	**0.015**	0.132	0.67	0.516	0.07	**0.707**	**0.007**
***IGF1R***	0.396	0.18	−0.259	0.39	0.549	0.055	0.580	0.04
***FGF7***	**0.791**	**0.001**	**0.632**	**0.02**	0.236	0.44	**0.880**	**0.0001**
***FGFR1***	**0.934**	**<0.0001**	**0.621**	**0.02**	**0.621**	**0.02**	**0.735**	**0.004**
**EMT and migration**								
***VIM***	**0.802**	**0.001**	0.165	0.60	0.571	0.04	**0.685**	**0.015**
***CDH1***	−0.055	0.86	−0.42	0.15	0	1	0.204	0.50
***ZEB2***	**0.885**	**<0.001**	0.446	0.11	0.434	0.14	**0.865**	**0.0001**
***SNAIL1***	0.472	0.11	−0.258	0.40	−0.357	0.23	−0.231	0.44
***SLUG***	**0.718**	**0.007**	0.228	0.450	0.318	0.286	**0.804**	**0.001**
***RUNX3***	0.462	0.11	0.562	0.05	−0.143	0.64	**0.602**	**0.03**
***CXCL12***	**0.907**	**<0.001**	0.581	0.04	**0.719**	**0.005**	**0.642**	**0.02**
**Cell proliferation and migration**								
***MMP2***	**0.698**	**0.008**	0.289	0.34	0.269	0.34	**0.690**	**0.01**
***MMP9***	0.203	0.50	0.400	0.18	−0.341	0.25	0.522	0.01
***MKI67***	−0.259	0.40	−0.204	0.51	−0.171	0.58	−0.147	0.88
***P53***	0.253	0.40	0.209	0.49	−0.297	0.32	0.569	0.045
***P16***	0.019	0.95	−0.132	0.66	−0.187	0.54	0.153	0.61
**Epigenetic**								
***EZH2***	−0.280	0.36	−0.368	0.21	−0.187	0.55	0.124	0.69
***HOTAIR***	−0.315	0.30	−0.569	0.04	0.022	0.94	−0.341	0.25
***H19***	0.25	0.42	0.30	0.32	−0.011	0.98	0.52	0.07
***DNMT1***	−0.23	0.45	−0.13	0.66	−0.34	0.25	−0.03	0.93
***DNArepair*** ***BRCA1***	−0.467	0.10	−0.357	0.23	−0.247	0.42	−0.163	0.59

**^a^** Spearman‘s rank test. Values in bold are statistically significant at a confidence level greater than 99% (*p* value < 0.01) and r < 0.6.

**Table 4:** Correlations between the expression of hormone receptors and genes involved in signaling pathways in intestinal GC.

Genes	*ERα*		*ERβ*		*AR*		*PR*	
	r	*p*-Value ^a^	r	*p*-Value ^a^	r	*p*-Value ^a^	r	*p*-Value ^a^
***ERα***	1	**< 0.0001**	**0.675**	**0.005**	**0.771**	**0.001**	**0.755**	**0.001**
***ERβ***	**0.675**	**0.005**	1	<0.0001	0.274	0.31	0.303	0.25
***AR***	**0.771**	**0.001**	0.274	0.31	1	<0.0001	0.846	<0.0001
***PR***	**0.755**	**0.001**	0.303	0.25	**0.846**	**<0.0001**	1	<0.0001
***ERRγ***	0.274	0.03	0.321	0.23	0.115	0.67	0.335	0.20
***GPER***	**0.871**	**<0.0001**	**0.621**	**0.01**	**0.771**	**0.001**	**0.846**	**<0.0001**
***AhR***	−0.011	0.68	−0.144	0.59	−0.018	0.69	−0.221	0.41
**Growth factors**								
***IGF1***	**0.794**	**0.0004**	0.339	0.20	**0.962**	**<0.0001**	**0.962**	**<0.0001**
***IGF1R***	**0.601**	**0.015**	0.140	0.61	**0.602**	**0.015**	0.497	0.06
***FGF7***	**0.819**	**0.0001**	0.322	0.22	**0.964**	**<0.0001**	**0.924**	**<0.0001**
***FGFR1***	**0.713**	**0.002**	0.227	0.34	**0.895**	**<0.0001**	**0.892**	**<0.0001**
**EMT and migration**								
***CDH1***	−0.353	0.20	−0.034	0.90	−0.308	0.24	−0.429	0.09
***VIM***	**0.592**	**0.02**	0.037	0.89	**0.821**	**0.0002**	**0.728**	**0.002**
***ZEB2***	**0.813**	**0.0001**	0.349	0.18	**0.907**	**0.0001**	**0.779**	**0.004**
***SNAIL1***	0.365	0.16	0.090	0.72	0.322	0.22	0.319	0.23
***SLUG***	0.440	0.08	−0.135	0.64	**0.631**	**0.01**	0.422	0.10
***RUNX3***	−0.068	0.80	0.360	0.17	−0.404	0.12	**0.699**	**0.003**
***CXCL12***	**−0.772**	**0.001**	−0.270	0.31	**0.921**	**<0.0001**	**0.834**	**<0.0001**
**Cell proliferation and migration**								
***MMP2***	0.605	0.01	0.076	0.78	**0.831**	**<0.0001**	**0.699**	**0.003**
***MMP9***	−0.087	0.75	−0.061	0.82	−0.302	0.26	−0.450	0.07
***MKI67***	**−0.691**	**0.003**	−0.350	0.18	**−0.771**	**<0.0001**	**−0.794**	**0.0001**
***p53***	**−0.789**	**0.0004**	−0.459	0.07	**−0.734**	**0.002**	**−0.625**	**0.01**
***p16***	0.284	0.23	0.044	0.88	0.442	0.09	0.411	0.11
***Epigenetic***								
***EZH2***	−0.526	0.04	−0.135	0.62	**−0.718**	**0.04**	**−0.818**	**0.0001**
***HOTAIR***	−0.152	0.57	0.049	0.86	−0.060	0.82	−0.057	0.83
***H19***	**0.830**	**0.0001**	0.49	0.054	**−0.880**	**<0.0001**	**0.82**	**0.0002**
***DNArepair*** ***BRCA1***	−0.059	0.83	0.131	0.63	−0.025	0.93	−0.258	0.33

**^a^** Spearman’s rank test (correlation between two quantitative parameters). Values in bold are statistically significant at a confidence level greater than 99% (*p* value < 0.01) and r < 0.6.

**Table 5:** Correlations between the expression of membrane receptors, *ERRγ* and *GPER,* in diffuse and intestinal GCs.

	(A) (Diffuse GC)				(B) (Intestinal GC)			
Genes	*ERRγ*		*GPER*		*ERRγ*		*GPER*	
	r	*p*-Value ^a^	r	*p*-Value ^a^	r	*p*-Value ^a^	r	*p*-Value ^a^
**Hormone receptors**								
***ERα***	−0.434	0.14	−0.011	0.97	0.274	0.03	**0.871**	**<0.0001**
***ERβ***	−0.499	0.08	−0.311	0.30	0.321	0.23	**0.621**	**0.01**
***AR***	0.099	0.75	0.480	0.09	0.115	0.67	**0.771**	**0.001**
***PR***	−0.370	0.21	−0.007	0.98	0.335	0.20	**0.846**	**<0.0001**
***ERRγ***	**1**	**<0.0001**	−0.567	0.04	1	<0.0001	0.494	0.005
***GPER***	−0.567	0.04	**1**	**<0.0001**	0.494	0.05	**1**	**<0.0001**
***AhR***	0.309	0.30	−0.085	0.78	**−0.617**	**0.01**	−0.405	0.14
**Growth factors**								
***IGF1***	−0.291	0.33	−0.103	0.74	0.188	0.48	**0.912**	**<0.0001**
***IGF1R***	0.396	0.18	0.013	0.96	0.182	0.50	0.561	0.02
***FGF7***	−0.555	0.05	−0.419	0.15	0.112	0.68	**0.838**	**<0.0001**
***FGFR1***	−0.156	0.12	0.044	0.88	−0.044	0.87	**0.727**	**0.001**
**EMT and migration**								
***CDH1***	−0.253	0.37	−0.066	0.83	−0.550	0.03	−0.362	0.17
***VIM***	0.214	0.48	−0.146	0.63	−0.339	0.20	0.506	0.05
***ZEB2***	0.264	0.38	−0.030	0.91	0.05	0.85	**0.709**	**0.002**
***SNAIL1***	−0.298	0.32	−0.569	0.04	−0.379	0.15	0.238	0.37
***SLUG***	−0.266	0.38	−0.266	0.53	−0.398	0.13	0.223	0.40
***RUNX3***	−0.308	0.31	−0.338	0.26	−0.284	0.28	−0.310	0.24
***CXCL12***	0.270	0.37	0.239	0.44	0.182	0.50	**0.741**	**0.001**
**Cell proliferation and migration**								
***MMP2***	**−0.720**	**0.01**	−0.333	0.27	−0.284	0.28	0.530	0.03
***MMP9***	−0.104	0.73	0.528	0.06	−0.5	0.08	−0.317	0.23
***MKI67***	0.011	0.93	−0.393	0.18	−0.424	0.10	**−0.797**	**0.0002**
***p53***	−0.35	0.24	−0.25	0.41	−0.262	0.29	**−0.711**	**0.002**
***p16***	−0.22	0.46	−0.44	0.14	**−0.646**	**0.007**	0.225	0.40
**Epigenetic**								
***EZH2***	0.077	0.80	−0.415	0.16	−0.419	0.11	**−0.727**	**0.001**
***HOTAIR***	0.172	0.58	−0.006	0.98	0.292	0.27	−0.118	0.66
***H19***	−0.39	0.19	−0.34	0.26	0.16	0.54	**0.820**	**0.0002**
***DNMT1***	−0.17	0.57	−0.56	0.049	−0.50	0.049	−0.54	0.033
***BRCA1***	−0.116	0.71	−0.422	0.15	**−0.671**	**0.005**	−0.288	0.28

**^a^** Spearman’s rank test (correlation between two quantitative parameters). Values in bold are statistically significant at a confidence level greater than 99% (*p* value < 0.01) and r < 0.6.

**Table 6:** Relationship between expression of genes involved in epigenetic regulation with clinical parameters in diffuse (**A**, n = 13) and intestinal (**B**, n = 16) GCs.

**(A)**	***EZH2***	***H19***	***HOTAIR***	***DNMT1***
**Gender,** **Male (n = 6)** **Female (n = 7)**	*p* = 0.73 2.11 (1.2–3.8) 2.5 (1.7–3.4)	*p* = 0.23 4.86 (1.1–42.3) 1.8 (1.2–6.2)	*p* = 0.23 3.9 (0–21.5) 36 (0–52.6)	*p* = 0.86 1.4 (1–1.7) 1.5 (1–1.7
**Age** **<60 years (n = 8)** **>60 years (n = 5)**	***p*** ** = 0.03** **2.12 (1.2–2.7)** **3.1 (1.7–3.8)**	*p* > 0.999 3 (1.1–6.2) 1.8 (1.2–42.3)	*p* = 0.12 2.58 (0–42) 19.5 (4–52)	*p* = 0.10 1.34 (1–1.7) 1.6 (1.3–1.7
**Tumor invasion** **T1-T2 (n = 2)** **T3-T4 (n = 11)**	*p* = 0.15 3.16 (2.9–3.4) 2.24 (1.2–3.8)	*p* = 0.31 1.53 (1.3–1.8) 3.54 (1.1–42.3)	***p*** ** = 0.013** **51.7 (51–52.6)** **4.1 (0–42.3)**	*p* = 0.14 1.7 (1.6–1.7) 1.4 (1–1.7)
**Vascular invasion** **negative (n = 3)** **positive (n = 10)**	***p*** ** = 0.049** **1.4 (1.2–2.2)** **2.7 (1.5–3.8)**	*p* = 0.29 1.77 (1.07–3.8) 3 (1.2–42.3)	*p* = 0.81 3.7 (1.4–36.1) 14 (0–52.6)	*p* = 0.39 1.33 (1.02–1.6) 1.52 (1–1.75)
**Lymphatic invasion** **negative (n = 1)** **positive (n = 12)**	*ND* 1.38 2.6 (1.2–3.8)	*ND* 3.83 2.4 (1.07–42.3)	*ND* 1.44 14 (0–52.6)	*ND* 1 1.52 (1–1.75)
**Peritoneal metastasis** **negative (n = 9)** **positive (n = 4)**	*p* = 0.26 2.7 (1.2–3.8) 1.9 (1.4–2.8)	*p* = 0.82 2.48 (1.07–43.3) 2.8 (1.2–6.2)	*p* = 0.50 4.1 (0–52.6) 27.8 (1.4–42.3)	*p* = 0.08 1.17 (1–1.6) 1.33
**TNM** **I-II (n = 5)** **III-IV (n = 8)**	*p* = 0.17 2 (1.2–2.7) 2.8 (1.4–3.8)	*p* = 0.72 2.48 (1.07–5.9) 2.82 (1.2–42.3)	***p*** ** = 0.03** **0 (0.2–21.5)** **27.8 (1.4–52.6)**	*p* = 0.75 1.36 (1.1–1.68) 1.55 (1–1.75)
**(B)**	***EZH2***	***H19***	***HOTAIR***	***DNMT1***
**Gender,** **Male (n = 7)** **Female (n = 9)**	*p* = 0.41 3.55 (1.4–5.5) 5.54 (1.1—9.4)	*p* = 0.83 11.1 (0.4–27.8) 5.4 (0.98–25.1)	*p* = 0.54 13.7 (2.5–62.1) 22.4 (0.2–67.8)	*p* = 0.35 1.6 (0.9–2.5) 2.1 (1.2–2.5)
**Age** **< 60 years (n = 1)** **> 60 years (n = 15)**	ND 1.46 5.1 (1.1–9.4)	ND 25.8 5.4 (0.4–25.1)	ND 62.1 19.2 (0.2–27.8)	ND 1.31 2.1 (0.9–2.5)
**Tumor invasion, T** **T1-T2 (n = 4)** **T3-T4 (n = 12)**	*p* = 0.13 5.81 (5.1–6.1) 3.51 (1.1–9.4)	***p*** ** = 0.013** **1.1 (0.4–2.9)** **10.97 (1–27.8)**	*p* = 0.40 18.1 (0.25–28.5) 20.8 (2.5–67.8)	*p* = 0.77 1.8 (1.4–2.4) 2.1 (0.9–2.5)
**Vascular invasion,** **Negative (n = 6)** **Positive (n = 10)**	*p* = 0.63 5.66 (1.1–9.5) 3.55 (1.4–9.5)	*p* = 0.71 4.2 (1.6–25.1) 9.64 (0.4–27.8)	*p* = 0.49 16.5 (0.2–61) 25.1 (2.5–67.8)	*p* = 0.95 2.13 (1.2–2.5) 1.99 (0.9–2.5)
**Lymphatic invasion,** **Negative (n = 10)** **Positive (n = 5)**	***p*** ** = 0.005** **5.53 (3.3–9.4)** **1.46 (1.1–3.5)**	***p*** ** < 0.001** **2.2 (0.4–10.8)** **15.9 (11.1–27.8)**	*p* = 0.86 20.8 (0.2–67.8) 28.2 (2.5–62.1)	***p*** ** = 0.05** **2.2 (1.4–2.5)** **1.3 (1.2–2.4)**
**Peritoneal metastasis** **negative (n = 15)** **positive (n = 1)**	ND 5.1 (1.1–9.4) 1.46	ND 5.4 (0.4–25.1) 25.8	ND 19.2 (0.2–67.8) 62.1	ND 2.1 (0.9–2.5) 1.31
**TNM** **I-II (n = 11)** **III-IV (n = 5)**	***p* = 0.013** **5.53 (1.4–9.4)** 1.46 (1.1–3.5)	***p*** ** = 0.002** **2.87 (0.4–12.5)** **15.9 (11.1–27.9)**	*p* = 0.83 19.2 (0.2–67.8) 28.1 (2.5–62.1)	*p* = 0.14 2.15 (0.9–2.5) 1.32 (1.2–2.4)

Median (range) of gene mRNA expression levels; *p*-value (Mann–Whitney). Significant *p* value in bold. ND = not determined.

## Data Availability

The original contributions presented in this study are included in the article material. Further inquiries can be directed to the corresponding author.

## Supplementary Materials

The following supporting information can be downloaded at: https://www.mdpi.com/article/10.3390/biomedicines13081815/s1, Table S1A: Statistical analysis of mRNA expression of steroid receptors in diffuse gastric cancer relative to the peri tumoral tissues.; Table S1B: Statistical analysis of mRNA expression of genes in intestinal gastric cancer relative to the peri tumoral tissues.
