# Supplementary material for: Involvement of Hormone Receptors, Membrane Receptors and Signaling Pathways in European Gastric Cancers Regarding Subtypes and Epigenetic Alterations: A Pilot Study
Source: Biomedicines. 2025 Jul 24;13(8):1815. doi: 10.3390/biomedicines13081815 (PMC12383840; doi:10.3390/biomedicines13081815)
Supplement: Supplementary file 1 [file biomedicines-13-01815-s001.zip › biomedicines-3673223-supplementary.pdf]

Table S1A: Statistical analysis of mRNA expression of steroid receptors in diffuse gastric cancer relative to the peri tumoral tissues.

Genes	Peri tumoral gastric tissues (n=11)[b]	ADCI (n=13)[b]	*p*-value[a]	% underexpression relative to peri tumoral tissues	% overexpression relative to peri tumoral tissues	*p*-value[c]
**A, Receptors (n=6)**						
ERα	1 (0.48-2.08)	**1.90 (0.64-3.16)**	**0.01**	0	15.4	0.54 (NS)
ERβ	1 (0.00-6.51)	2.68 (0.68-4.44)	0.21 (NS)	0	46.2	0.23 (NS)
PR	1 (0.51-4.53)	1.43 (0.62-2.74)	0.16 (NS)	0	0	0.93 (NS)
AR	1 (0.54-2.54)	0.94 (0.26-1.58)	0.19 (NS)	7.7	0	0.93 (NS)
ERRγ	1 (0.04-4.77)	**0.07 (0.01-1.77)**	**0.011**	**7.7**	0	**0.043**
GPER	1 (0.13-2.00)	**0.08 (0.04-0.38)**	**<0.0001**	**84.6**	0	**0.015**
**B, Epigenetic (n=4)**						
EZH2	1 (0.21-2.19)	**2.51 (1.19-3.81)**	**<0.0001**	0	23.1	0.14 (NS)
HOTAIR	0 (0.00-3.08)	**8.40 (0.00-52.60)**	**0.001**	0	0	-
H19	1 (0.38-12.3)	**2.48 (1-42.3)**	**0.047**	0	46	0.23 (NS)
DnmT1	1 (0.77-1.47)	**1.5 (1-1.75)**	**0.001**	0	0	**0.04**
**DNA repair (n=1)**						
BRCA1	1 (0.31-1.70)	**2 (1.47-3.33)**	**<0.0001**	0	7.7	0.36 (NS)

[a] Mann Whitney test.

[b] Median (range) of gene mRNA levels.

[c] Chi-Square test.

Table S1B: Statistical analysis of mRNA expression of genes in intestinal gastric cancer relative to the peri tumoral tissues.

Genes	Peri tumoral gastric tissues (n=11)[b]	ADK (n=13)[b]	*p*-value[a]	% underexpression relative to peri tumoral tissues	% overexpression relative to peri tumoral tissues	*p*-value[c]
**A, Receptors (=6)**						
**ERα**	1 (0.48-2.08)	0.69 (0.17-1.22)	**0.043**	12.5	0	0.64 (NS)
**ERβ**	1 (0.00-6.51)	0.40 (0.12-2.57)	0.06 (NS)	31.3	0	0.11 (NS)
**AR**	1 (0.54-2.54)	0.25 (0.04-0.76)	**<0.0001**	**56.3**	0	**0.0085**
**PR**	1 (0.51-4.53)	0.48 (0.03-3.22)	**0.011**	37.5	6.3	0.068 (NS)
**ESRRG**	1 (0.04-4.77)	0.03 (0.00-0.18)	**0.0002**	**100**	0	**0.0003**
**GPER**	1 (0.13-2.00)	0.06 (0.02-0.15)	**<0.0001**	**100**	0	**0.0003**
**B, Epigenetic (n=4)**						
**EZH2**	1 (0.21-2.19)	4.34 (1.11-9.45)	**<0.0001**	0	**75**	**0.0006**
**HOTAIR**	0 (0.00-3.08)	20.8 (0.25-67.84)	**<0.0001**	-	-	-
**H19**	1 (0.38-12.3)	6.94 (0.4-27.8)	**0,039**	0	**56**	0.069 (NS)
**DnmtT1**	1 (0,77-1,47)	2.05 (0.9-2.5)	0,0002	0	0	0,33 (NS)
**C, DNA repair (n=1)**						
**BRCA1**	1 (0.31-1.70)	3.12 (1.38-6.86)	**<0,0001**	0	**56.3**	**0.007**

[a] Mann Whitney test.

[b] Median (range) of gene mRNA levels.

[c] Chi-Square test.
